# Demonstration of the Effect of Generic Anatomical Divisions versus Clinical Protocols on Computed Tomography Dose Estimates and Risk Burden

**DOI:** 10.1371/journal.pone.0097691

**Published:** 2014-05-30

**Authors:** Rachael E. Moorin, David A. J. Gibson, Rene K. Forsyth, Richard Fox

**Affiliations:** 1 Centre for Population Health Research, Curtin University, Perth, Western Australia, Australia; 2 Centre for Health Services Research, School of Population Health, University of Western Australia, Perth Western Australia, Australia; 3 Department of Medical Imaging Science, Curtin University, Perth, Western Australia, Australia; 4 School of Physics, University of Western Australia, Perth, Western Australia, Australia; Technische Universitaet Muenchen, Germany

## Abstract

**Objective:**

Choosing to undertake a CT scan relies on balancing risk versus benefit, however risks associated with CT scanning have generally been limited to broad anatomical locations, which do not provided adequate information to evaluate risk against benefit. Our study aimed to determine differences in radiation dose and risk estimates associated with modern CT scanning examinations when computed for clinical protocols compared with those using anatomical area.

**Methods:**

Technical data were extracted from a tertiary hospital Picture Archiving Communication System for random samples of 20–40 CT examinations per adult clinical CT protocol. Organ and whole body radiation dose were calculated using ImPACT Monte Carlo simulation software and cancer incidence and mortality estimated using BEIR VII age and gender specific lifetime attributable risk weights.

**Results:**

Thirty four unique CT protocols were identified by our study. When grouped according to anatomic area the radiation dose varied substantially, particularly for abdominal protocols. The total estimated number of incident cancers and cancer related deaths using the mean dose of anatomical area were 86 and 69 respectively. Using more specific protocol doses the estimates rose to 214 and 138 incident cancers and cancer related deaths, at least doubling the burden estimated.

**Conclusions:**

Modern CT scanning produces a greater diversity of effective doses than much of the literature describes; where a lack of focus on actual scanning protocols has produced estimates that do not reflect the range and complexity of modern CT practice. To allow clinicians, patients and policy makers to make informed risk versus benefit decisions the individual and population level risks associated with modern CT practices are essential.

## Introduction

Over the four decades since the introduction of Computed Tomography (CT) scanning, diagnostic imaging technological advancements have been a potent factor driving innovations in medicine [1]. Recent concerns about the radiation dose associated with CT scanning have led to guidelines, advising on clinical indications for utilisation and/or reference levels for radiation dose received from each type of examination[2]–[4]. Recent studies reporting the cancer risks of ionizing radiation [5]–[9] have spurred intense debate about the risks of diagnostic imaging, and how these risks ought to be incorporated into the decision making process[8]–[12].

Large scale data capture of CT scanner radiation dose in databases have been proposed to enable institutional benchmarking, optimization of CT protocols, and quality control [9], [13]. These data will also enable more accurate patient specific dose estimation than was possible from previously available data sources, enabling more accurate patient specific risk assessment to better inform imaging decisions [9], [14].

Estimates of radiation dose and cancer risk from CT scanning were classically undertaken using ‘typical’ anatomically based CT protocol/machine settings[5], [15]–[17]. However, the enormous technological advance in CT scanning has brought diversification in examination types (protocols) within anatomical locations subsequently affecting the radiation dose and risk [8], [9]. Reliance on simplistic anatomical location based radiation dosimetry and derived risk estimates that do not adequately reflect current practice limits the ability of referring clinicians to make informed risk: benefit decisions for their patients.

The aim of our study was to examine the radiation dose associated with modern CT scanning examinations by anatomical location versus actual clinical protocol to demonstrate the degree of variation in radiation dose and its impact on population burden and risk estimates.

## Materials and Methods

### Study Design and Setting

A cross-sectional, observational, retrospective study design with technical CT data collected from a large metropolitan tertiary/teaching hospital in Western Australia (using two 64-slice CT machines of the same make and model), via the Picture Archiving Communication System (PACS), on adult diagnostic CT scanning protocols (excluding extremities) identified by way of discrete protocol code/naming conventions used by the institution. Prior to collection of the data the study was approved by the Western Australian Department of Health Human Research Ethics Committee, with a waiver of informed consent for the retrospective review of electronic medical records.

### Data Sources

A random sample of 20 to 40 cases from each protocol identified was collected on scans performed between 1^st^ January and 30^th^ April 2011. Rarely fewer than twenty cases were identified within the collection period; in this event all cases were included in the study. If the technical parameters appeared particularly variable up to forty cases were collected. A sample of 20 cases is at least double studies using self-report data have used [15], comparable to similar studies [9] and conforms to the European guidelines on the sample required to assess usual patient doses [18]. Protocol information (excluding the scout view) consisted of separate scanning sequences (phases) whenever present. The technical parameters collected included kilovoltage (kV), milliamperage (mA), tube rotation times, collimation widths, pitch, scanning method, anatomical reference start-stop positions, volume weighted CT dose index (CTDIvol) and dose–length product (DLP).

### Radiation Dosimetry

Values of CTDIvol (inclusive of automated tube current modulation) and DLP were used to calculate the organ specific dose (mGy) and effective dose (mSv) for each sequence using the ImPACT dosimetry calculation software [19]. This tool allows organ and effective dose to be estimated in a population of patients based on Monte Carlo simulation in an idealized phantom. Justification of this method and its limitations are presented in the discussion section. Scan length was obtained by dividing DLP by CTDIvol present in each case’s dose report. Where ImPACT gave slightly different values of CTDIvol to those reported by the machine, the effective dose given by ImPACT was corrected by the ratio of the reported CTDIvol and the ImPACT estimate. Cumulative protocol values of CTDIvol, DLP, organ dose and effective dose were calculated by summation of all sequences reported for each case. The mean, minimum and maximum values for each parameter were reported for each protocol and anatomical area.

### Cancer Risk Modelling

The age and gender specific lifetime attributable risk (LAR) inferable from a single exposure was estimated for the mean, lowest and highest dose across protocols and each anatomical area. This was achieved using the protocol specific organ dose and the age/sex-specific risk coefficients from tables 12D-1 and 12D-2 of the BEIR VII report [20]. The LAR of cancer incidence and mortality resulting from radiation dose to the remainder and ‘other’ organs was calculated using doses for organs not named in the BEIR VII LAR tables but have a weight by International Commission on Radiological Protection (ICRP) 103 [21], or are included in the remainder organs by ICRP 103, and weighting them by the risk attributed by BEIR VII for ‘other’ organs. This method assumes all such organs contribute equally to risk. The analysis was repeated for all combinations of age (ranging from 18 to 80 years) in yearly increments, using linear interpolation of the BEIR VII risk coefficients from the two nearest tabulated ages when data were not available for a specific age. To estimate the cancer incidence risk and cancer related mortality attributable to CT scanning for persons aged over 80 years the linear interpolation was extended until the estimated number of cancers or mortality reached zero for each protocol using the same method described above. The age and gender specific count of CT scanning procedures performed in Western Australia obtained from Medicare Australia and WA PACS data were used to estimate the number of incident cancers and cancer related mortality attributable to CT.

## Results

### Radiation Dose Associated with CT Protocols

Table 1 shows a summary of data recorded for each CT scanning protocol in the study. There was wide variation in the radiation dosimetry associated with each protocol not completely attributable to the number of sequences. Protocols for temporal bone, sinuses (non-contrast), abdomen for renal colic, abdomen/pelvis, pelvis (non-contrast), chest/abdo/pelvis, thoracic and lumbar spine CT scanning consistently included only a single sequence. However, the maximum to minimum ratios indicated a large variation in radiation dose. Table 1 also presents the mean and range of radiation dose attributable to CT scanning according to anatomical area, ‘all protocols,’ where highly variable with maximum to minimum ratios ranging from 4 (CTDIvol for chest/abdo/pelvis) to 91 (DLP for Abdominal CT) were observed.

**Table 1:** Variation in the number of sequences, dose-length product (DLP) and volume weighted Computed Tomographic dose index (CTDIvol) recorded for selected CT scanning examinations performed in a Western Australian tertiary hospital over a three month period in 2011.

Anatomical location	Protocol Name	Number of cases	Sequences in protocol				Protocol DLP^1^				Protocol CTDIvol^1^			
			Mean	Min	Max	Max:Min	Mean	Min	Max	Max:Min	Mean	Min	Max	Max:Min
**Head**	Head (Non-contrast)	20	1.5	1	3	3	1207.8	842.3	2699.2	3	64.6	39.5	158.8	4
	Head (Contrast)	17	1.1	1	2	2	1064.9	633.0	1952.3	3	51.2	32.3	112.3	3
	Head Pre and Post Contrast	28	2.0	2	3	2	1928.4	1008.6	3603.5	4	104.3	45.1	166.4	4
	Angiogram Head	20	1.3	1	3	3	734.5	65.8	2027.6	31	37.2	3.8	122.0	33
	Temporal Bones	10	1.0	1	1	1	661.2	501.6	792.3	2	65.9	49.0	69.5	1
	**All protocols**	**95**	**1.4**	**1**	**3**	**3**	**1119.4**	**65.8**	**3603.5**	**55**	**64.6**	**3.8**	**166.4**	**44**
**Facial bones**	Facial bones (Non-contrast)	10	1.2	1	2	2	845.2	270.7	3050.0	11	34.5	16.1	115.7	7
	Facial bones (Contrast)	20	1.3	1	4	4	630.7	179.9	2139.2	12	34.7	7.4	130.8	18
	Sinuses (Non-contrast)	10	1.0	1	1	1	157.3	103.7	392.6	4	8.8	6.8	22.7	3
	Sinuses (Contrast)	10	1.1	1	2	2	506.7	345.8	1076.5	3	26.4	17.0	45.1	3
	Sinus and Brain (Non-contrast)	11	1.4	1	2	2	1705.7	391.6	3064.9	8	69.9	22.5	114.2	5
	Orbits and/or Brain (Non-contrast)	10	1.1	1	2	2	299.0	233.2	669.2	3	19.6	15.5	45.1	3
	Orbits and/or Brain (Contrast)	7	1.1	1	2	2	385.5	233.2	503.2	2	23.9	17.0	33.9	2
	**All protocols**	**78**	**1.2**	**1**	**4**	**4**	**647.1**	**103.7**	**3064.9**	**30**	**31.1**	**6.8**	**130.8**	**19**
**Soft tissue neck**	Neck (Non-contrast)	20	1.3	1	2	2	521.6	51.6	1526.9	30	17.7	2.8	50.3	18
	Neck (Contrast)	20	1.4	1	3	3	451.9	232.3	937.0	4	22.8	6.9	61.4	9
	Angiogram Neck (Carotid)	20	1.3	1	2	2	633.7	242.3	1579.5	7	23.3	5.9	66.1	11
	**All protocols**	**60**	**1.3**	**1**	**3**	**3**	**535.7**	**51.6**	**1579.5**	**31**	**21.3**	**2.8**	**66.1**	**24**
**Chest**	Chest High Res	20	2.1	1	4	4	488.1	131.1	1183.1	9	13.7	3.5	30.9	9
	Chest (Non-contrast)	20	1.2	1	4	4	418.6	135.6	1183.1	9	10.7	3.5	55.6	16
	Chest with Contrast	40	1.1	1	3	3	464.3	175.5	998.5	6	11.8	4.3	44.4	10
	Chest Pulmonary Embolism Study	20	3.6	3	7	2	738.2	41.8	1635.7	39	47.8	14.8	103.7	7
	Angiogram Thoracic Aorta	20	3.8	3	5	2	799.9	192.4	1596.1	8	36.8	20.9	52.3	3
	Angiogram Cardiac (non-coronary)	20	1.5	1	2	2	747.4	141.3	2407.9	17	32.6	9.1	68.5	8
	**All protocols**	**140**	**2.2**	**1**	**7**	**7**	**609.4**	**41.8**	**2407.9**	**58**	**25.6**	**3.5**	**103.7**	**30**
**Abdomen (+/− Pelvis)**	Abdomen Renal Colic	20	1.0	1	1	1	149.6	98.6	239.5	2	3.1	2.4	5.3	2
	Angiogram Renal	14	3.3	1	5	5	689.5	361.8	1358.7	4	47.0	15.1	120.1	8
	Abdomen and Pelvis (Non-contrast)	20	1.0	1	1	1	564.5	103.4	1169.1	11	11.0	2.4	22.5	10
	Abdomen and Pelvis (Contrast)	20	1.7	1	3	3	903.4	329.4	1786.7	5	19.7	6.4	39.0	6
	Virtual Colonoscopy	20	2.4	2	3	3	372.3	221.0	1360.5	6	7.4	4.7	25.3	5
	Angiogram Visceral	20	4.3	1	6	6	1895.1	39.6	3599.8	91	60.1	2.4	162.0	69
	**All protocols**	**114**	**2.3**	**1**	**6**	**6**	**762.4**	**39.6**	**3599.8**	**91**	**24.7**	**2.4**	**162.0**	**69**
**Pelvis only**	Pelvis (Non-contrast)	20	1.0	1	1	1	657.9	306.8	1189.1	4	21.3	8.8	35.0	4
	Pelvis (Contrast)	20	1.5	1	4	4	997.1	223.8	2036.5	9	29.2	5.5	64.8	12
	**All protocols**	**40**	**1.3**	**1**	**4**	**4**	**827.5**	**223.8**	**2036.5**	**9**	**25.2**	**5.5**	**64.8**	**12**
**Chest/Abdo/Pelvis**	Chest/Abdo/Pelvis (Non-contrast)	10	1.0	1	1	1	997.2	463.4	1816.7	4	14.1	7.5	25.2	3
	Chest/Abdo/Pelvis (Contrast)	10	1.0	1	1	1	933.1	307.4	1919.2	6	13.5	7.0	29.0	4
	**All protocols**	**20**	**1.0**	**1**	**1**	**1**	**965.2**	**307.4**	**1919.2**	**6**	**13.8**	**7.0**	**29.0**	**4**
**Spine**	Spine Cervical (Non-Contrast)	10	1.3	1	2	2	1117.5	210.5	2832.6	13	48.6	9.5	126.7	13
	Spine Thoracic (Non-Contrast)	10	1.0	1	1	1	1032.3	374.7	1787.9	5	28.8	9.6	48.1	5
	Spine Lumbar (Non-Contrast)	10	1.0	1	1	1	990.9	432.9	2480.4	6	24.4	16.4	36.7	2
	**All protocols**	**30**	**1.1**	**1**	**2**	**2**	**1046.9**	**210.5**	**2832.6**	**13**	**34.0**	**9.5**	**126.7**	**13**

1 Where applicable values are generated using summed value for cases where multiple sequences were recorded.

**Figure 1:**
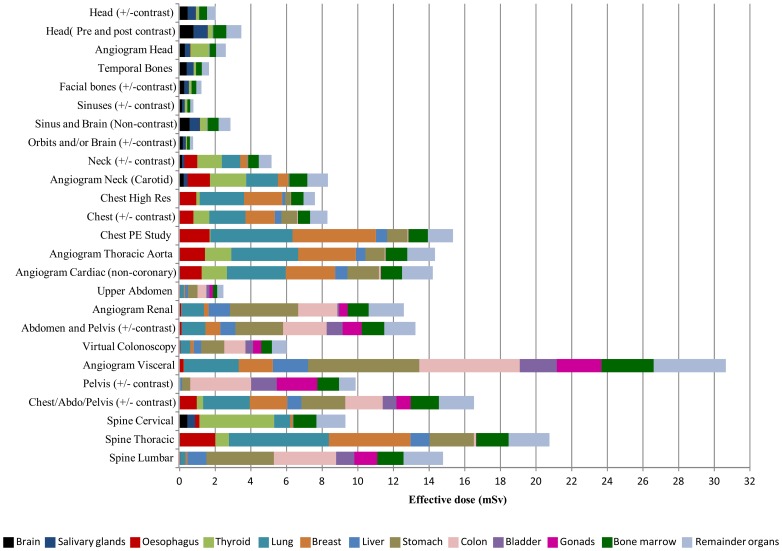
Mean whole body and organ specific effective doses (mSv) for selected CT scanning examinations performed in a Western Australian tertiary public hospital in 2011.

Figure 1 shows a summary of the mean total and organ specific effective doses attributable to cases collected for the CT scanning protocols included in the study. Note, for simplicity some protocols have been paired (+/− contrast) where no substantive differences were discernible resulting in 25 bars for the total 34 clinical protocols identified in Table 1. Visceral spiral (helical) angiography produced the highest mean effective whole body dose of 31.2 mSv, while CT of the sinuses produced the lowest mean effective dose (0.4 mSv). Of the 34 individual protocols, 11 (32%) produced mean effective doses greater than 10 mSv. Four protocols (12%) produced mean effective doses in excess of 15 mSv while two (6%) produced mean effective doses in excess of 20 mSv. Only seven protocols (20%) produced mean effective doses of less than 2 mSv. Only one protocol (visceral spiral angiography) produced a mean organ specific dose contribution greater than 6 mSv (6.2 mSv to the stomach). This protocol’s total effective dose also comprised 5.6 mSv from the colon and 4.1 mSv from the remainder organs. Thoracic spine CT was the only other protocol to include an organ specific dose contribution greater than 5 mSv (5.6 mSv from the lung). Several protocols produced specific organ contributions to effective dose between 4 and 5 mSv, these were: cardiac non-coronary spiral (helical) angiography (4.7 mSv from the breast and 4.6 mSv from the lung), CT of the thoracic spine (4.6 mSv from the breast), and CT of the cervical spine (4.2 mSv from the thyroid). When evaluating the effective doses according to anatomical area a wide variation was observed with the largest variation noted for abdominal protocols and the lowest variation in head closely followed by facial bone protocols.

### Variation in the LAR (Expressed as a Percentage) of Cancer Incidence and Cancer Related Mortality Across Anatomical Areas

**Figure 2:**
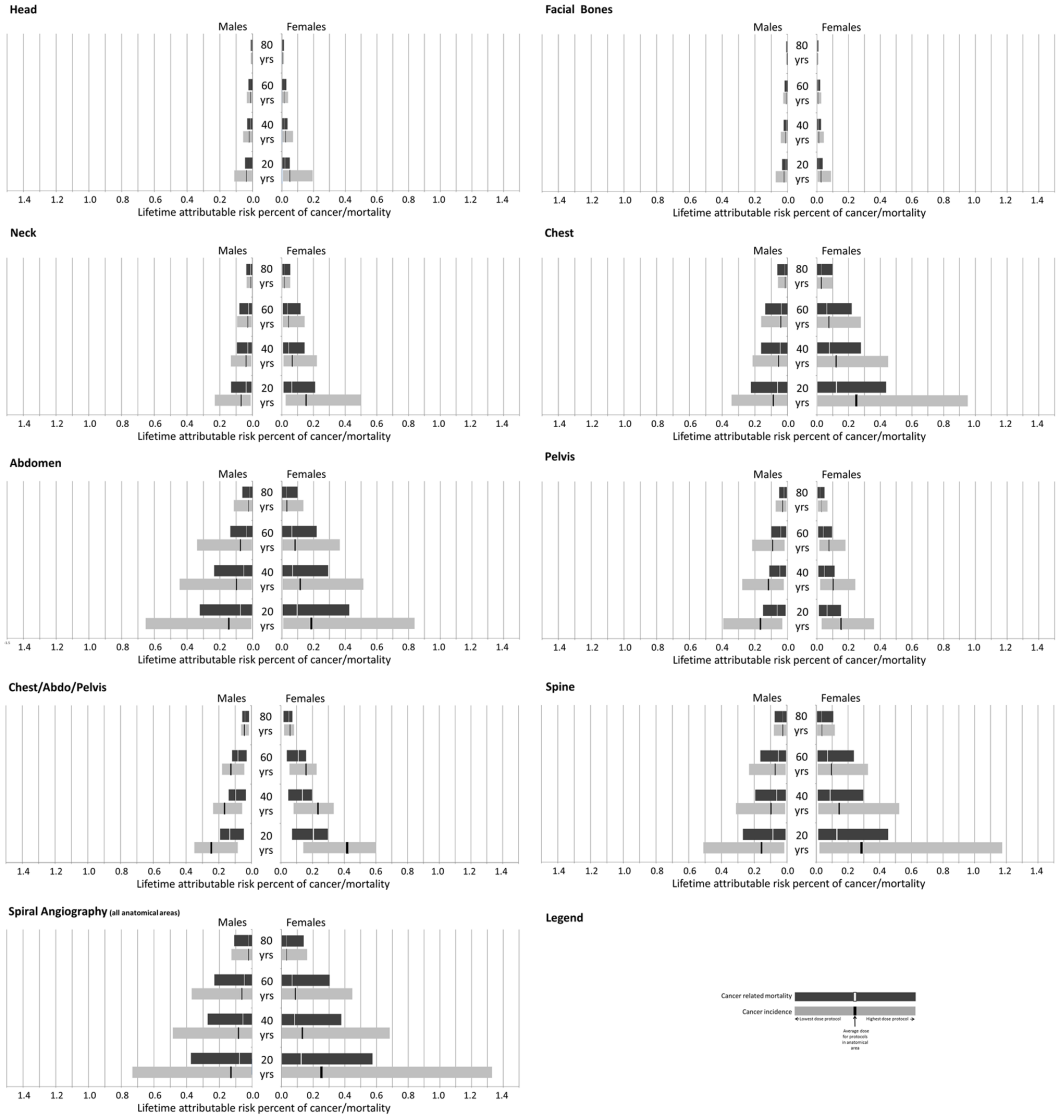
Variation in the lifetime attributable risk percent of cancer incidence (light bars) and mortality (dark bars) according to gender and age at a single exposure from CT scanning protocols grouped according to anatomical location.

As expected there was a large variation in the estimated risk of incident cancers and cancer related mortality across anatomical areas (Figure 2) driven by differences in the radio-sensitivity of the organs included within the scanning field, the radiation dose generated by the CT protocol and gender. The estimated risk of incident cancers and cancer related mortality was considerably higher in females compared with males, except for CT scanning of the pelvis where the estimated risk of cancer for females was consistently lower than males. The largest difference across genders was consistently observed for chest CT. Using the mean dose protocol, females at age 20 years had a 0.25% risk of cancer compared with 0.09% for males and a 0.12% risk of cancer related death compared with 0.06% for males. The magnitude of this difference is highly subject to the dose scenario evaluated, for example when the protocol giving the maximum dose was evaluated (end of the bars in Figure 2) females at age 20 years were estimated to have a 0.94% risk of incident cancers compared with 0.33% for males and a 0.43% risk of cancer related deaths compared with 0.22% for males.

When anatomical area mean dose was considered (black lines in Figure 2), facial bones CT was attributed the lowest risk of incident cancers and cancer related mortality while chest/abdo/pelvis CT was attributed the highest risk of incident cancers and cancer related mortality. This ranking of anatomical areas varied dependent on both the radiation dose scenario and to a lesser extent age at CT scan.

### Impact of the Dose Scenario on Burden of Cancer and Cancer Related Mortality Attributable to CT Scanning

Table 2 shows a demonstration of the number of incident cancers and cancer related mortality attributable to selected CT scanning examinations, using doses derived from anatomical area versus clinical protocols. If the mean dose derived from protocols within each anatomical area was used as the input to the risk modelling, 86 incident cancers and 69 cancer related deaths were estimated to be attributable to the independent effect (unadjusted for competing risk factors not considered in the BEIR VII risk model) of CT scanning radiation dose. However, using actual protocol doses the estimates rose to 214 and 138 incident cancers and cancer related deaths, an increase of a factor of 2.5 and 2.0 respectively. The range of predicted cancers was highly variable for anatomical areas comprising multiple protocols with four anatomical areas providing estimates varying by over 10 (cancers) predicted between the anatomical area estimate and individual protocol estimates. The largest difference in the anatomical area and protocol estimates was in abdomen CT. Large maximum to minimum ratios were also observed in six anatomical areas, with four having a maximum to minimum ratios of greater than 50, one exceeding 600 (abdomen).

**Table 2:** Estimated number of incident cancers and cancer related mortality attributable to selected CT scanning undertaken in WA during 2010/11 and 2011/12 using protocol and anatomical area based risk modelling.

Anatomical location	CT scanning protocol	Incident cancers					Cancer related mortality				
		n	min^1^	max^2^	range^3^	max:min	n	min^1^	max^2^	range^3^	max:min
**Head**	Head (+/− Contrast)	12.1					7.2				
	Temporal Bones	0.8					0.5				
	Angiogram Head	0.1					0.1				
	**Average Head**	**4.3**	**0.1**	**12.1**	**11.9**	**95.5**	**2.6**	**0.1**	**7.2**	**7.2**	**102.1**
**Facial bones**	Facial bones OR Sinuses (+/− Contrast)	2.5					1.4				
	Sinus and Brain (Non-Contrast)	0.7					0.4				
	Orbits and/or Brain (+/− Contrast)	0.1					0.0				
	**Average Sinuses/Facial bones**	**1.1**	**0.1**	**2.5**	**2.4**	**40.0**	**0.6**	**0.0**	**1.4**	**1.4**	**38.6**
**Soft tissue neck**	Neck (+/− Contrast)	3.8					2.7				
	Angiogram Neck (Carotid)	0.6					0.5				
	**Average Soft tissue neck**	**2.2**	**0.6**	**3.8**	**3.2**	**6.2**	**1.6**	**0.5**	**2.7**	**2.2**	**6.0**
**Chest**	Chest (+/− Contrast) including High Res	19.5					15.6				
	Chest PE Study	3.8					3.0				
	Angiogram Thoracic Aorta	0.6					0.5				
	Angiogram Cardiac (Non-Coronary)	0.2					0.2				
	**Average Chest**	**6.0**	**0.2**	**19.5**	**19.3**	**100.0**	**4.8**	**0.2**	**15.6**	**15.5**	**100.7**
**Abdomen**	Abdomen +/− Pelvis (+/− Contrast)	63.4					38.9				
	Virtual Colonoscopy	0.2					0.1				
	Angiogram Renal	0.1					0.1				
	Angiogram Visceral	0.9					0.6				
	**Average Abdomen**	**16.2**	**0.1**	**63.4**	**63.3**	**679.4**	**9.9**	**0.1**	**38.9**	**38.8**	**619.9**
**Pelvis**	Pelvis (+/− Contrast)	1.8					0.9				
	**Average Pelvis**	**1.8**	**1.8**	**1.8**	**0.0**	**1.0**	**1.8**	**1.8**	**1.8**	**0.0**	**1.0**
**Chest/Abdo/Pelvis**	Chest/Abdo/Pelvis (+/− Contrast)	38.4					28.0				
	**Average Chest/Abdo/Pelvis**	**38.4**	**38.4**	**38.4**	**0.0**	**1.0**	**38.4**	**38.4**	**38.4**	**0.0**	**1.0**
**Spine**	Spine Cervical (+/− Contrast)	9.6					5.7				
	Spine Thoracic (+/− contrast)	3.2					2.4				
	Spine Lumbar (+/− Contrast)	40.8					22.4				
	Spine Multiple Areas (+/− Contrast)	2.4					1.4				
	**Average Spine**	**14.0**	**2.4**	**40.8**	**38.4**	**16.9**	**8.0**	**1.4**	**22.4**	**21.1**	**16.6**
**Spiral Angiography** 4	Angiogram Head	0.1					0.1				
	Angiogram Neck (Carotid)	0.6					0.5				
	Angiogram Thoracic Aorta	0.6					0.5				
	Angiogram Cardiac (Non-Coronary)	0.2					0.2				
	Chest PE Study	3.8					3.0				
	Angiogram Renal	0.1					0.1				
	Angiogram Visceral	0.9					0.6				
	Angiogram Unspecified	7.2					5.1				
	Angiogram Cardiac (Coronary)	1.3					1.0				
	**Average Spiral Angiography**	**1.6**	**0.1**	**7.2**	**7.1**	**76.8**	**1.2**	**0.1**	**5.1**	**5.0**	**80.6**
**Total**	**Based on anatomical areas**	**85.6**			**128.3**	**2.5**	**68.8**			**69.6**	**2.0**
	**Based on protocols**	**213.9**					**138.4**				

n = Number of estimated incident cancers/cancer related mortality based on cancer risk modelling from the mean radiation dose of the cases collected in protocols included in the study.

1 number based on the minimum dose protocol within the anatomical area.

2 number based on the maximum dose the protocol within the anatomical area.

3 Range = number based on the maximum dose protocol - number based on the minimum dose protocol.

4 Spiral angiography also included within each anatomical location for the public providers. Note separation of spiral angiography codes are not available for private providers except coronary angiography and hence is excluded.

## Discussion

Decisions regarding whether to undertake a particular diagnostic imaging test should always be made balancing risks against potential benefits. Given the there may be strategies which may incur lower radiation dose while still affording an acceptable level of diagnostic accuracy for the particular clinical circumstance, comprehensive information regarding radiation dose and risk associated with modern CT scanning is required. This is particularly important in patients where the magnitude of the risk burden from ionising radiation is high (ie children, young adults and women). Our study has provided comprehensive information about the radiation dose and risk burden of modern CT scanning in order to facilitate incorporation into clinical decision making.

This study has demonstrated modern CT practice comprises multiple protocols, across most anatomical areas, resulting in a wide range of radiation doses. This variation in radiation dose interacted substantially with the lifetime attributable risk ascribed to ionising radiation, greatly influencing the number of incident cancers and cancer related mortality estimated to be associated with CT scanning. The effective radiation dose, and subsequent risk, resulting from a single CT examination is highly dependent on machine parameters (ie the protocol used) and the radiosensitivity of the anatomical area scanned. Both of these factors need to be considered when dosimetric and risk assessments are undertaken. Using anatomical area for dose and risk estimates does not accurately account for the significant differences between protocols. The end result is an inaccurate perception of risk to individual patients but also the impact of CT radiation burden at a population level.

The majority of authors when reporting CT radiation dose either state a broad range (eg 10 to 100 mGy [22]) or provide a series typical doses for each anatomical area [23], while some authors have quantified differences in effective dose produced in limited scenarios by changing certain technical parameters [24]. However, only a few studies have previously published limited range of protocol specific dose observations, either using survey data or directly collected from PACS dose reports [8], [9]. Our study has also demonstrated variation in intra-protocol dose suggesting patient specific modification of technical settings occurs, most likely based on either clinical requirements or patient habitus. This finding is welcome in response to concerns in the literature over the lack of modification of standard protocols with respect to body habitus and thus potential over-exposure of patients [4]. While we cannot definitively determine the intra-protocol variation observed in our study was due to patient characteristics, it is a plausible explanation for our observations.

A limitation of our study is that our method of estimating effective and organ dose did not include size specific dose estimation (SSDE) methods since information regarding the body habitus of the patients included in the study were not available. However, for this study the use of SSDE would not change the (numerical) deviation between the two methods because it concerns the same patient groups. In addition, while it is very important to account for patient size when estimating individual patient radiation dose [25], effective dose is intended to represent the dose to a population of patients (as we have done in our study) not individual patient dose [26]. This is an important distinction since effective dose is derived from measurements in an idealized phantom that integrates the relative weighting of the radiosensitive organs exposed and does not reflect the morphometrics of an individual patient [26]. All estimates of radiation dose have limitations, for example SSDE does not take into account variations in dose based on variations in scan length, assumes patients are centred in the CT gantry so that magnification effects are minimized and cannot be used for estimation of organ dose, and thus cannot be used to estimate effective dose [26]. Thus while SSDE is recommended and appropriate for estimating individual patient radiation dose it is not suitable when organ and effective dose estimates are required and is not necessary when estimating the average radiation dose characteristics of a particular examination ie examination specific rather than patient specific dosimetry is required.

In our study we aimed to estimate the average effective radiation dose for each adult CT protocol using a random sample of adult patients. The use of random sampling methodology was used to capture any underlying variation in doses produced for each scanning protocol, comparable to other published research [9], and avoid recall or selection bias associated with the use of survey methods. Additionally, the use of actual scan parameters and dosimetry information recorded at the time of imaging rather than reliance on self-selected mean doses, ‘standard’ protocols or phantoms facilitate a more accurate representation of actual dose in practice. Our data source and sampling method provide a more rigorous picture of real CT practice, rather than idealised or theoretical CT doses and practices.

Detailed information regarding patient numbers according to CT protocols from routinely captured administrative data has allowed for cancer and cancer related mortality attributable to CT scanning to be estimated, for individual protocols and by anatomical area. The risk estimates reported in this study are not based on epidemiological data of actual malignancies in populations of patients receiving CT scans (such information is unavailable). The estimates are extrapolations of the attributable cancer risk models developed in the BEIR VII report [20] using standard Monte Carlo methods modelling photon transport in CT. This study employs previously used methods to estimate risk and are the best available given available data [16]. Our estimation of the number of incident cancers and cancer related mortality attributable to CT scanning assumed all providers would give identical doses. Similar assumptions have been used in these types of estimates previously [27]. However, the focus of this study was not to estimate the actual risk of cancer and cancer related mortality but to demonstrate the impact on risk estimates from inter-protocol variation versus aggregation of CT protocols to anatomical areas.

While the BEIR VII report provides a framework for estimating age, sex and organ specific cancer risks from a radiation exposure it does not fully account for underlying pathology and life expectancy. The BEIR VII risks should be considered representative of the independent effect of radiation dose and can only be said to account for competing risks included in the original BEIR VII models. The estimated number of incident cancers and cancer related mortality are presented here as a demonstration of the magnitude of the effect on risk estimates of using protocol rather than anatomic area dose. There is substantial difficulty in estimating population cancer risk as noted by the International Organization for Medical Physics (IOMP) [28]. In our study the imprecision is equally applied to both dose scenarios (anatomical and protocol) hence the magnitude of the effect of using a simplistic generalised method (ie anatomic based) over a more comprehensive and clinically realistic model (protocol based) is not affected by the concerns of the IOMP. These concerns primarily rest with debate regarding acceptance of the ‘linear, no-threshold theory’ for ionising radiation exposure risk. The linear, no-threshold theory forms the foundation for radiation protection recommendations by international and national committees [20], [21], [29]. Criticism of the linear, no-threshold theory rest on statistical uncertainty for the relationship between radiation exposure and cancer incidence at low doses (less than 100 mSv) [12], [21]. However, current biological evidence does not support a threshold model where exposure to sub-100 mSv radiation doses represent no risk [20], [29], [30]. Additionally, other studies estimating the cancer incidence resulting from the independent effects of CT radiation exposure have been published using the linear, no-threshold theory and BEIR-VII LAR estimates [6], [8]. Therefore our study has employed conservative, clinically representative, peer-reviewed and internationally recognised methodology for dose and risk estimation.

### Conclusion

Radiation dose and risks associated with CT scanning have commonly been presented using broad anatomical locations without consideration for the diversity of modern CT examinations performed within each region. This leaves referring clinicians and patients with limited or simplistic information to evaluate risk against benefit. Our study demonstrated the insufficiency of presenting radiation dose according to anatomical areas, rather than specific CT protocols, using rigorous CT technical data sources and established risk estimation methods. Lack of focus on actual clinical scanning protocols has produced dose estimates that do not reflect current clinical practice, therefore to improve risk versus benefit decision making differentiation of the associated radiation dose resulting from the variety of services present in modern CT is essential.
